# The Effects of Sand Incorporation on the Pore Structure, Strength, and Fractal Characteristics of Alkali-Activated Slag Cementitious Materials

**DOI:** 10.3390/ma18122797

**Published:** 2025-06-13

**Authors:** Yuchen Ye, Zhenyuan Gu, Yi Wang, Ying Sun, Chenhui Zhu, Jie Yang

**Affiliations:** School of Transportation and Civil Engineering, Nantong University, Nantong 226019, China; jstxyeyuchen@outlook.com (Y.Y.); guzhenyuan0507@ntu.edu.cn (Z.G.); wang12yi@ntu.edu.cn (Y.W.); ying.sun1993@ntu.edu.cn (Y.S.)

**Keywords:** alkali-activated slag concrete, fractal theory, pore structure, sand content

## Abstract

Sand content plays a critical role in regulating the structural compactness and strength development of alkali-activated slag cementitious materials. In this study, three types of specimens—pure slag paste, standard sand mortar, and fine sand mortar—were prepared to investigate the effects of sand incorporation on pore structure and fractal characteristics. Mechanical properties, pore structure, and micro-morphology were systematically evaluated at different curing ages. Mercury intrusion porosimetry (MIP) was employed to measure porosity, pore size distribution, and the threshold pore diameter, while fractal dimensions were calculated to quantify pore complexity and compactness. The results showed that the pure slag paste achieved the highest compressive strength at all ages but posed environmental concerns due to high resource consumption. In contrast, sand-incorporated mortars exhibited stable strength development and continuous pore structure refinement. Notably, the use of fine sand in Group C reduced slag content by approximately 5.6% compared to Group A, contributing to lower CO_2_ emissions and enhanced sustainability. Fractal analysis revealed a strong correlation between fractal dimension, pore compactness, and compressive strength. A higher fractal dimension indicated a more complex and interconnected pore network, promoting matrix densification. At 90 days, Group C achieved the highest fractal dimension and lowest porosity, attributed to the micro-filling effect of fine sand, which facilitated the formation of a denser and more continuous gel network. These findings provide a theoretical foundation for the multiscale characterization of alkali-activated cementitious systems and support the design of more sustainable mix formulations.

## 1. Introduction

Ordinary Portland cement concrete (OPCC) is among the most widely used construction materials globally, extensively employed in infrastructure such as roads, bridges, and buildings. With the rapid pace of urbanization and continued infrastructure expansion, the demand for OPCC increases year by year. However, its production relies heavily on the high-temperature calcination of limestone and clay, resulting in substantial natural resource depletion and significant carbon dioxide (CO_2_) emissions. It is estimated that approximately 1 ton of CO_2_ is released for every ton of cement produced, amounting to around 1.35 billion tons annually—roughly 7% of global anthropogenic greenhouse gas emissions. This energy-intensive and emission-heavy process poses serious ecological and environmental challenges, highlighting the urgent need for sustainable, low-carbon alternatives.

In recent years, alkali-activated slag concrete (AASC) has gained considerable attention as a promising eco-friendly cementitious material. AASC utilizes industrial by-products such as slag, fly ash, and kaolinite, which are activated by alkaline solutions to form clinker-free cementitious systems primarily composed of aluminosilicate gels. Compared to OPCC, AASC offers comparable or even superior compressive strength, early-age performance, and durability. By repurposing industrial waste and eliminating the need for traditional calcination, AASC significantly reduces both natural resource consumption and CO_2_ emissions, offering notable economic and environmental advantages [1,2,3,4,5]. As a result, AASC is increasingly regarded as an ideal low-carbon alternative for sustainable construction.

Nevertheless, existing studies have reported variability in the performance of AASC, particularly with regard to its long-term mechanical stability [6]. This variability is largely attributed to the physical and chemical inconsistencies of raw materials such as slag and fly ash. For instance, the microcracks generated during mechanical crushing can lead to cumulative internal structural damage. Therefore, a detailed investigation of AASC’s microstructure—especially the formation mechanisms and evolution of pore structures—is essential for understanding its mechanical behavior, optimizing mix proportions, and enhancing long-term durability.

Several techniques are currently employed to characterize the pore structure of cementitious materials. Low-field nuclear magnetic resonance (NMR) offers the precise detection of micropores smaller than 10 nm but has difficulty identifying macropores larger than 1 μm [7]. Isothermal desorption is effective for analyzing micropores and mesopores in the 0.5–35 nm range but is inadequate for characterizing pores larger than 100 nm [8]. Micro-computed tomography (micro-CT) provides a high-resolution visualization of macropores at the micron scale, yet it has limited ability to resolve nanoscale pores and is not widely applicable to cement-based materials [9,10,11,12]. Mercury intrusion porosimetry (MIP), widely favored for its broad detection range (0.005–750 μm) and high resolution, enables the acquisition of multidimensional pore parameters—including porosity, pore size distribution, and threshold pore diameter—and is thus considered a primary technique for characterizing the pore structure of cementitious systems.

However, the wide pore size distribution and irregular pore morphology in AASC limit the ability of traditional geometric parameters—such as porosity and average pore diameter—to adequately capture pore complexity and spatial heterogeneity. As a result, fractal theory, which effectively describes irregular and complex systems, has been increasingly applied to the study of pore structures in cementitious materials. Fractal dimensions quantitatively represent pore complexity, uniformity, and compactness and are generally found to correlate positively with material performance.

Previous research has identified fractal characteristics in the micropores, fracture surfaces, and interfacial regions of concrete [13,14,15,16,17,18,19]. Cleaves et al. [20] confirmed the fractal nature of porous materials using scanning electron microscopy, while Eibeck et al. [21] and Zhang et al. [22] investigated the fractal dimensions of cracks and pore networks in concrete. Jin et al. [23] further examined the fractal behavior of fracture surfaces in AASC subjected to freeze–thaw cycles. Collectively, these studies validate the effectiveness of fractal theory in accurately characterizing complex microstructures and in predicting the macroscopic properties of cementitious systems.

Additionally, mathematical models such as the Menger sponge—which structurally resembles the principles underlying MIP measurements—have been employed to construct fractal geometric representations of pore networks [24,25,26]. Multifractal analysis further enhances the understanding of pore evolution and its relationship to material performance [27]. By integrating MIP data with fractal theory, it becomes possible to achieve a multidimensional characterization of AASC pore structures and to establish intrinsic correlations between microstructure and macroscopic behavior.

Unlike previous studies that focus exclusively on slag paste or standard sand mortar, the present work uniquely investigates the combined effects of incorporating both standard and fine sands into AASC. It addresses a critical research gap by systematically examining how varying sand particle sizes influence microstructural evolution, pore characteristics, and related fractal properties. These insights contribute to more refined strategies for material optimization and performance enhancement.

## 2. Materials and Methods

### 2.1. Materials and Mix Proportions

**Figure 1:**
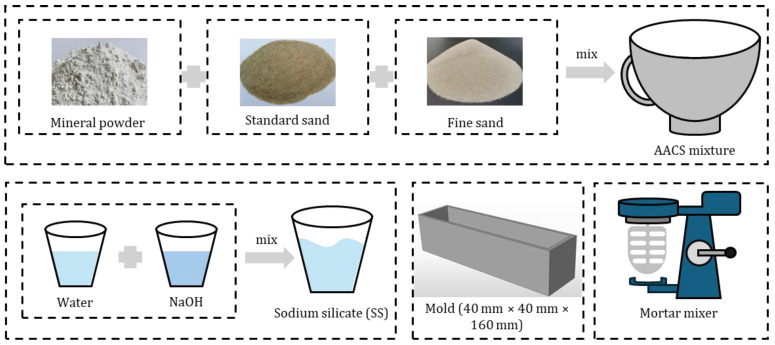
Raw materials.

The materials used in this study include slag powder, fine sand, water glass, standard sand, and mixing water. The chemical composition of the slag powder, supplied by Hunan Huaxin Xianggang Cement Co., Ltd. (Tanxiang, China), is listed in Table 1. Fine sand was sourced from Liuhe River in Nanjing and sieved to a particle size range of 0.08–0.63 mm. Water glass (WG), manufactured by Nanjing Chemical Factory (Nanjing, China), had a solid content of 27.43%, with its chemical composition presented in Table 2. Prior to use, the water glass was diluted with tap water and mixed with analytically pure sodium hydroxide (AR grade, Shanghai Shiyi Chemical Reagent Co., Ltd., Shanghai, China) to prepare an alkaline activator solution with a modulus of 1.40. Standard sand, conforming to Chinese ISO specifications, was used in both drying shrinkage and autogenous shrinkage tests for alkali-activated slag cementitious mortar. Its particle size ranged from 0.5 to 1.0 mm, in accordance with GB/T 17671-2021 [28]. Laboratory tap water was used as the mixing water. The raw materials used in this study are illustrated in Figure 1.

The specimen grouping for alkali-activated slag cementitious materials is presented in Table 3. Group A consisted of pure alkali-activated slag paste, while Groups B and C contained alkali-activated slag mortars. Specifically, Group C incorporated 22.5 g of fine sand, selected based on optimal particle packing density principles to enhance the micro-filling effect [29]. This approach aims to improve matrix compactness and mechanical performance, as supported by previous studies [30].

### 2.2. Experimental Methods

#### 2.2.1. Mechanical Performance Testing

**Figure 2:**
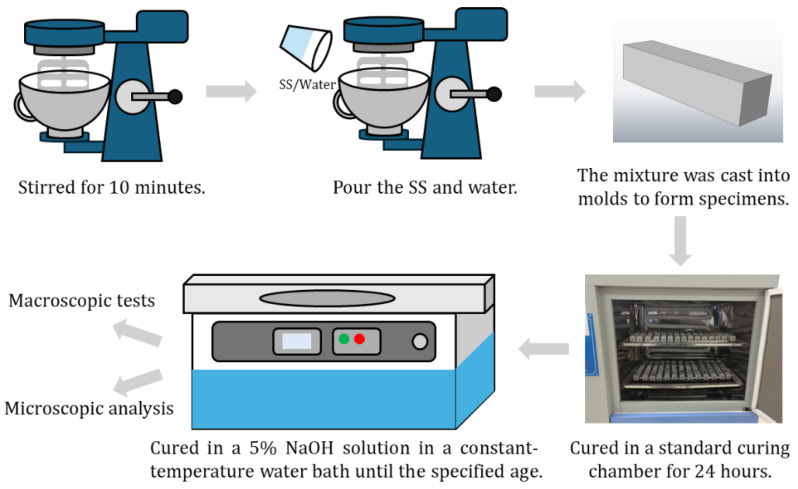
Experimental flowchart.

Compressive strength tests for alkali-activated slag cementitious mortar were performed in accordance with GB/T 17671-2021, Cement Mortar Strength Testing Method (ISO Method), using prism specimens with dimensions of 40 mm × 40 mm × 160 mm. For pure paste specimens, smaller prisms measuring 20 mm × 20 mm × 80 mm were used, with a fixed water-to-binder ratio of 0.3. The alkaline activator was water glass with a modulus of 1.4, and the Na_2_O content was maintained at 6% of the total mass of active cementitious materials. The total mixing water was adjusted to account for the water introduced by the water glass, ensuring consistency with the target water-to-binder ratio. Mortar flowability was controlled within the range of 180–190 mm. After casting, specimens were cured in molds within a standard curing chamber for 24 h, then demolded and transferred to a thermostatic water bath containing a 5% NaOH solution. This curing method, commonly employed in alkali-activated slag systems, was selected for its proven effectiveness in sustaining the hydration of slag particles under alkaline conditions. Compared to other methods, such as sealed or pure water curing, immersion in 5% NaOH enhances hydration kinetics and promotes greater structural densification, as validated by previous research [29]. A schematic of the experimental procedure is provided in Figure 2.

#### 2.2.2. Autogenous Shrinkage Test

**Figure 3:**
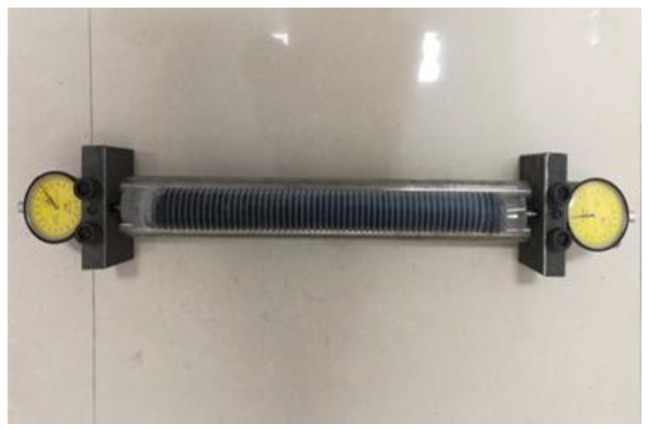
Autogenous shrinkage test apparatus.

The autogenous shrinkage of alkali-activated slag mortar was measured in accordance with ASTM C1698-09 [31], using polyethylene corrugated tube molds. Figure 3 shows the actual test setup, including the corrugated tube, fixed support frame, and dial gauge for axial deformation measurement. This method converts the volumetric change in the material into axial displacement by leveraging the high axial flexibility of the corrugated tube, allowing for the continuous and accurate monitoring of early-age shrinkage while minimizing external influences such as gravity, temperature variations, and mold restraint.

Although the low initial stiffness of the paste may introduce some mold-related influence within the first hour after casting, this effect becomes negligible after approximately 10 h, once the material reaches its final set. In this study, three specimens were prepared for each group and cast into polyethylene corrugated tubes with an internal diameter of 33 mm. The sealed tubes were horizontally mounted on a steel frame, and axial deformation was recorded using dial gauges starting from the initial setting time.

The autogenous shrinkage strain was calculated as follows:

(1) $$\varepsilon =\frac{∆L}{L}\times 100\%$$

where ∆*L* is the length change in the specimen, and *L* is the length of the paste.

#### 2.2.3. MIP Testing

Mercury intrusion porosimetry (MIP) is a widely adopted technique for characterizing porous materials, particularly effective for detecting open pores ranging from 5 nm to 200 μm in cement-based composites. Compared to other methods, MIP exhibits superior sensitivity to larger pores, making it particularly valuable for evaluating properties related to material strength and durability. In this study, pore structure measurements were conducted using a PoreMaster GT60 (Anton Paar, Graz, Austria) high-performance automated mercury porosimeter, operating under pressures from 0.007 MPa to 350 MPa (1 psi to 50,000 psi) with a mercury contact angle set at 140°. The detailed MIP testing procedure follows the methodology described in the authors’ previously published work, “Strength Characteristics and Microstructure Analysis of Alkali-Activated Slag–Fly Ash Cementitious Material” [32].

### 2.3. Fractal Model Establishment

Alkali-activated slag cementitious materials typically possess complex and heterogeneous pore structures across multiple scales, which significantly influence their compactness and mechanical performance [32]. Traditional geometric parameters—such as porosity and average pore diameter—are insufficient to fully capture the structural complexity of and spatial variability in these pores. In contrast, fractal dimensions provide a more accurate quantitative assessment of pore complexity, connectivity, and spatial distribution, offering a superior means of characterizing microstructural features. As such, fractal theory has become a valuable analytical tool for establishing correlations between pore-scale characteristics and macroscopic material behavior. By effectively capturing the intricacy of pore networks, fractal dimension enables a more precise evaluation of how microstructure governs the mechanical properties of alkali-activated systems. In this study, fractal analysis is integrated with MIP data to explore the multiscale structural features of AASC, enabling a deeper understanding of strength evolution mechanisms.

**Figure 4:**
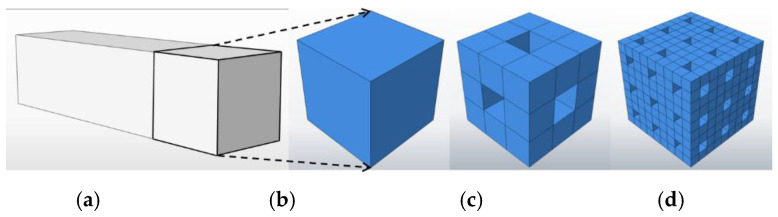
Menger sponge model: (**a**) original block, (**b**–**d**) Menger sponge model.

Among various fractal models—such as the Sierpinski carpet and multifractal approaches—the Menger sponge, first proposed by Menger in 1926, is selected in this study due to its structural similarity to the hierarchical and nested pore networks commonly observed in alkali-activated slag cementitious materials. While multifractal models can capture more heterogeneous systems, the Menger sponge offers a simpler yet sufficiently representative geometric framework that closely aligns with the pore size distributions measured by MIP testing. Constructed through iterative geometric operations, the Menger sponge effectively simulates highly intricate spatial configurations, making it particularly well-suited for characterizing multiscale, nested pore networks. The pore structure of alkali-activated slag cementitious materials exhibits a sponge-like morphology and spatial arrangement, closely resembling the Menger sponge architecture. This resemblance makes the Menger sponge model a practical and well-matched choice for fractal analysis in this context. Figure 4 illustrates the conceptual basis of this model: Figure 4a shows a representative cubic region extracted from the hardened specimen, while Figure 4b presents the corresponding Menger sponge unit. The fractal dimension of the Menger sponge model is calculated as follows:

The initial cube with side length L is subdivided into m^3^ smaller cubes, each with side length l = L/m. A small number a of these cubes are randomly removed, leaving m^3^-a cubes. This subdivision and removal process is repeated iteratively. After k iterations, the remaining number of cubes is (m^3^ − a)^k^, and the smallest unit side length becomes l_k_ = L/m^k^.

The total volume of the remaining smaller cubes *V*_t_ after *t* iterations is as follows:

(2) $$V_{t}=N_{t}l_{t}^{3}=\frac{l_{t}^{3-D_{M}}}{L^{{-D}_{M}}}$$

where *N_t_* is the number of remaining cubes after t iterations, lt is the dimension of cubes after *t* iterations, *L* is the initial side length, and *D_M_* is the fractal dimension.

The total number of cubes *N_t_* is as follows:

(3) $$N_{t}={\left(\frac{l_{t}}{L}\right)}^{{-D}_{M}}$$

Thus, the pore volume of the Menger sponge model can be expressed as the following:

(4) $$V=L^{3}-V_{t}=L^{3}-\frac{l_{t}^{3-D_{M}}}{L^{{-D}_{M}}}$$

Differentiating with respect to *l_k_* yields the pore volume distribution density:

(5) $$f\left(l_{t}\right)=-\frac{dV}{dl_{t}}=\frac{3-D_{M}}{L^{{-D}_{M}}}l_{t}^{2-D_{M}}$$

Taking logarithms on both sides gives the following:

(6) $$lgf\left(l_{t}\right)=\left(2-D_{M}\right)lgl_{t}+lg(\frac{3-D_{M}}{L^{{-D}_{M}}})$$

Fractal dimensions were calculated based on MIP data to quantitatively assess pore complexity and compactness. To ensure the robustness of the results, a standard mercury contact angle of 140° was applied, and all calculations followed well-established methodologies. Furthermore, comparative analyses were conducted to evaluate the sensitivity of the fractal dimension results to common MIP limitations, including intrusion pressure hysteresis and ink-bottle effects. These evaluations confirmed minimal bias and demonstrated the high reliability of the derived fractal dimensions.

## 3. Results and Discussion

### 3.1. Strength Development of Alkali-Activated Slag Cementitious Materials

**Figure 5:**
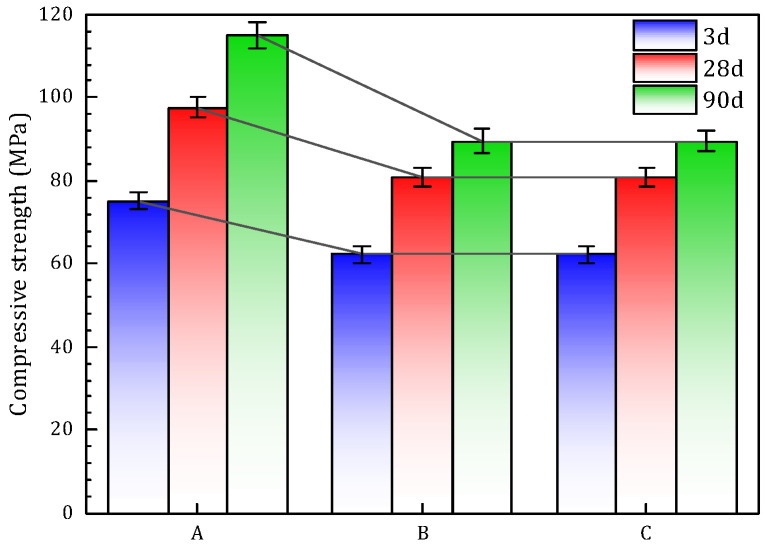
Compressive strength development of specimens.

Figure 5 presents the compressive strength development of the different specimen groups. All samples exhibited a progressive increase in compressive strength from 7 to 90 days. Group A consistently achieved the highest strength values across all curing ages. At 7 days, Group A reached 75.07 MPa, significantly surpassing Group B (62.2 MPa) and Group C (60.8 MPa). By 28 days, Group A maintained its lead with a strength of 97.5 MPa, while Groups B and C recorded comparable values of 80.9 MPa and 80.8 MPa, respectively. Continued hydration up to 90 days further improved performance: Group A attained 114.85 MPa, remaining the strongest overall, while Group C increased notably to 95.36 MPa, exceeding Group B’s 89.59 MPa.

The superior performance of Group A is attributed to rapid early hydration and extensive gel formation, particularly evident at early ages. In contrast, Groups B and C—both incorporating sand—showed initially lower strengths but demonstrated stable and continuous growth over time. Notably, Group C ultimately outperformed Group B, likely due to the enhanced micro-filling effect provided by the finer sand particles.

These strength development trends suggest that while Group A offers the highest overall mechanical performance, Groups B and C present a viable balance between performance and sustainability. The reduction in slag content—especially in Group C—contributes to environmental benefits without severely compromising strength, highlighting its promising potential for practical applications in green construction.

### 3.2. Autogenous Shrinkage Test Results

The test results showed that the autogenous shrinkage strain of all specimens remained below 1618 × 10^−6^ mm/mm, indicating a consistently low level of internal shrinkage throughout the curing period. Given this moderate shrinkage and the absence of external drying conditions, it is unlikely that the observed increase in threshold pore diameter in the specimens at 90 days was solely attributable to autogenous deformation. Moreover, the MIP measurements were conducted in accordance with standardized protocols designed to minimize common artifacts such as pressure hysteresis and ink-bottle effects.

Therefore, the observed pore coarsening is primarily attributed to progressive microstructural degradation—most likely driven by localized gel densification, internal stress accumulation, and the development of microcracks over time. This interpretation is consistent with observations reported in previous studies on alkali-activated systems [33].

### 3.3. SEM Analysis

**Figure 6:**
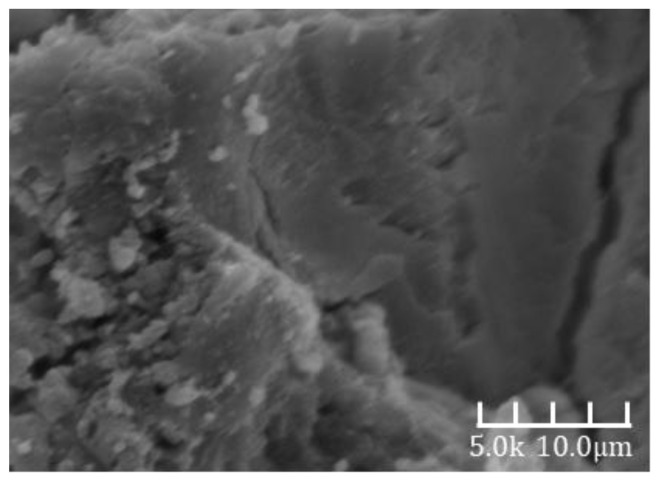
A 3d-A SEM image.

Figure 6 presents a scanning electron microscope (SEM) image of the alkali-activated slag cement paste from Group A at a hydration age of 3 days. The microstructure appears highly compact, with the hardened matrix primarily composed of dense gel phases and unreacted slag particles. The corresponding compressive strength at this age reached 75 MPa, indicating the development of a dense internal structure, which aligns well with the compact morphology observed in the SEM image.

**Figure 7:**
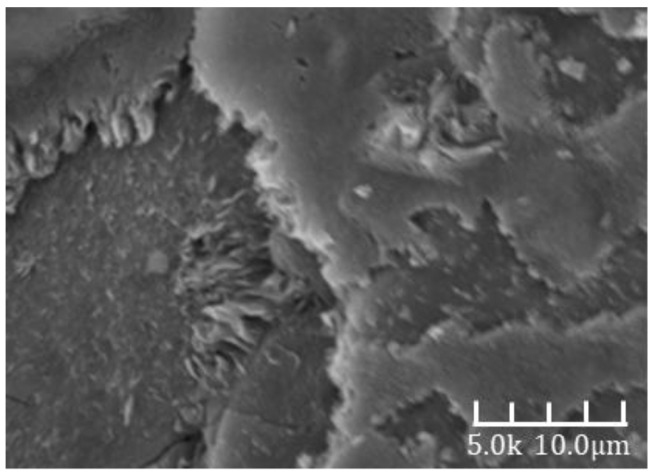
A 3d-B SEM image.

**Figure 8:**
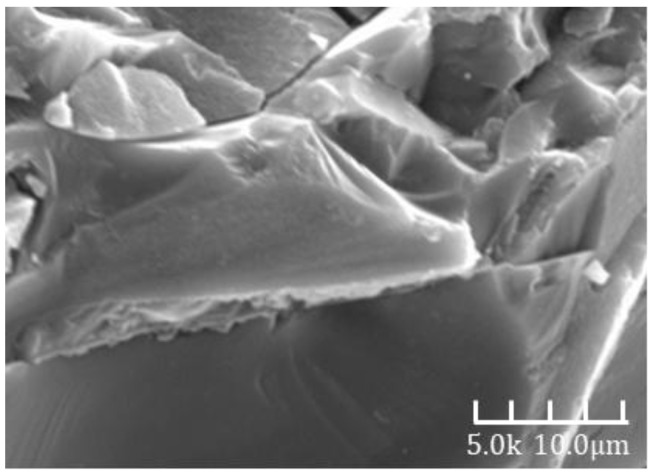
A 28d-B SEM image.

Figure 7 and Figure 8 show the SEM images of alkali-activated slag mortar from Group B at hydration ages of 3 days and 28 days, respectively. A clear comparison reveals that the hardened matrix in Figure 7 is significantly denser than that in Figure 6. The cementitious matrix exhibits strong interfacial bonding with the aggregates, which are tightly embedded within the hydration products. The aggregate surfaces are coated with dense hydration phases and display minimal porosity. These phases are well integrated with the surrounding matrix, forming a continuous and interconnected microstructure. This microstructural refinement is directly reflected in the macroscopic properties, as the 28-day compressive strength is approximately 30% higher than the 3-day value [32].

**Figure 9:**
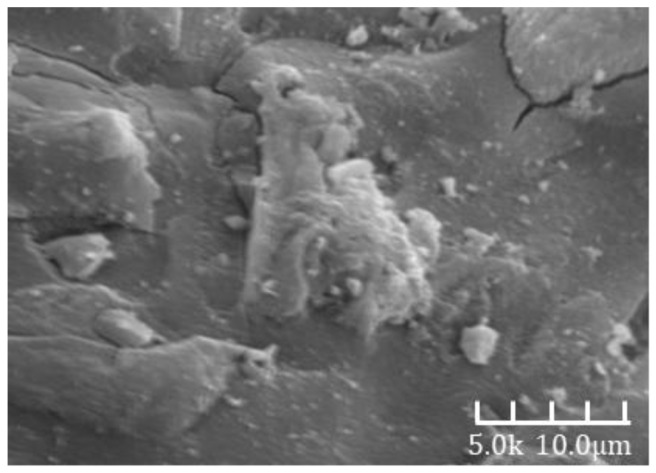
A 3d-C SEM image.

**Figure 10:**
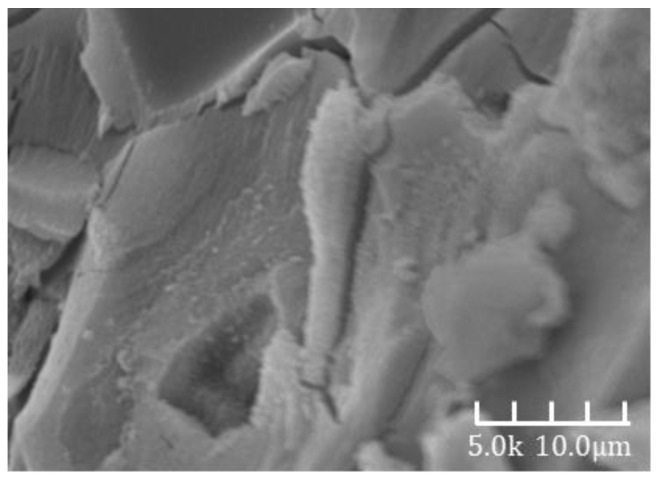
A 28d-C SEM image.

Figure 9 and Figure 10 show the SEM images of Group C alkali-activated slag mortar at hydration ages of 3 days and 28 days, respectively. A distinct difference is evident between the two images: the microstructure in Figure 9 is markedly denser than that in Figure 8. The cementitious matrix exhibits strong bonding with the fine aggregates, which are tightly encapsulated by compact hydration phases. Most surface voids are filled, and the aggregate surfaces are uniformly coated with dense products. These hydration phases are intricately interwoven with the surrounding matrix, forming a cohesive and continuous structure. This microstructural enhancement directly contributes to improved macroscopic performance, as the 28-day compressive strength is approximately 38% higher than that at 3 days [32].

Moreover, similar phenomena have been reported in prior studies investigating the long-term evolution of alkali-activated slag systems. Zhu et al. [32] and Zhang et al. [34] observed that prolonged curing can lead to the shrinkage of the C-(A)-S-H gel, particularly in binder-rich systems without inert fillers. This gel shrinkage may generate internal tensile stresses, which in turn promote the formation and propagation of microcracks. EDS analyses in these studies further revealed a decrease in calcium and silicon concentrations in certain localized regions after extended hydration, suggesting a gradual weakening of the gel structure. In the absence of aggregate-induced constraint, such as in Group A, this microstructural degradation can result in the coalescence of pores and the loss of matrix continuity, ultimately contributing to the observed increase in threshold pore diameter at 90 days. These insights align with our findings and support the interpretation that late-age pore coarsening in Group A is primarily driven by shrinkage-induced damage and progressive structural relaxation.

### 3.4. Characteristic Parameters of Pore Structure

**Figure 11:**
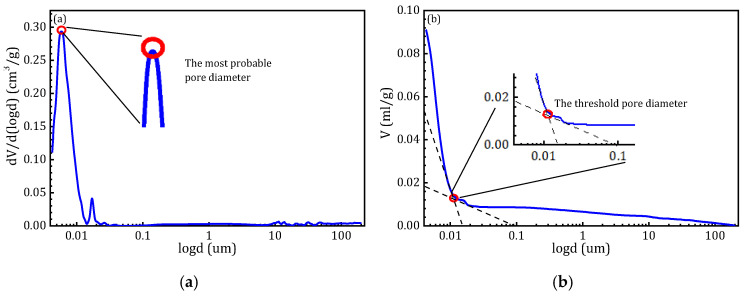
Methods for determining the most probable and threshold pore sizes of renewable micro-powders: (**a**) a method for determining the most probable pore size; (**b**) a method for determining the threshold pore size.

To illustrate the characteristics of pore size distribution, Figure 11 presents the methodology for determining the most probable pore diameter and the threshold pore diameter, using the 3-day specimen of Group A as an example. The most probable pore diameter refers to the pore size occurring the most frequently, typically represented by the peak of the differential pore size distribution curve. A larger most probable pore diameter generally indicates a shift toward coarser pores and an increase in average pore size [35]. Katz et al. [36] defined the threshold pore diameter as the inflection point on the cumulative intrusion curve, marking the onset of pore connectivity. In relatively homogeneous porous media—such as soils, rocks, or ordinary cement pastes—this parameter can often be directly identified as the point where the cumulative pore volume begins to rise sharply. However, in cementitious composites incorporating additives, the pore structure is more complex, and multiple abrupt increases may appear in the small pore size range, making direct identification challenging [37,38]. To address this, Figure 11a illustrates an alternative approach: tangents are drawn along the slow-rising and rapid-rising segments of the cumulative intrusion curve, with their intersection defined as the threshold pore diameter.

In addition to pore volume and pore size distribution, the MIP test also yields several other characteristic parameters that further describe the pore structure of the material. These key parameters are summarized in Table 4. The data in Table 4 reveal significant differences in pore structure parameters among specimens with varying compositions and hydration ages, consistent with previous analyses. Group A exhibited a marked decrease in threshold pore diameter from 3 to 28 days, indicating rapid early hydration and the formation of abundant gel phases that refined and sealed larger pores. However, by 90 days, the threshold pore diameter in Group A increased from 1415.26 nm to 2843.1 nm, suggesting the coarsening of the pore structure. This phenomenon is likely due to the shrinkage of the hydration gels during prolonged curing, which can induce microcracking and pore coalescence. Moreover, the absence of inert fillers such as sand renders the matrix more susceptible to internal stress accumulation, exacerbating cracking and compromising the integrity of the gel network over time. The influence of internal deformation was further evaluated through autogenous shrinkage testing, which showed strain values below 1618 × 10^−6^ mm/mm, confirming that the observed changes in threshold pore diameter were not caused by measurement artifacts from MIP but instead reflect true microstructural evolution.

In contrast, Group C demonstrated a continuous decrease in porosity and progressive pore structure refinement throughout the curing period, particularly at 90 days. This improvement is attributed to the micro-filling effect of fine sand, which not only reduces initial porosity but also stabilizes the internal matrix against shrinkage-induced damage. The fine particles effectively occupy voids between hydration products, promoting denser packing and more homogeneous gel formation. This densification enhances long-term structural integrity and mitigates the formation of connectivity pores or the propagation of microcracks.

For Group B, the threshold pore diameter steadily increased with hydration age, reaching 7382.83 nm at 90 days. This trend aligns with SEM observations showing widening interfacial cracks, indicating the ongoing expansion of interface pores during prolonged curing, thereby reducing overall matrix compactness. In contrast, Group C exhibited a significant reduction in threshold pore diameter to 1877.2 nm at 90 days. This improvement is directly linked to the micro-filling capacity of fine sand, which effectively filled internal paste voids and enhanced the compactness of the aggregate–matrix interface.

Regarding the most probable pore diameter, all specimens exhibited a decreasing trend with prolonged hydration, eventually stabilizing around 4 nm. At 90 days, Group C achieved the smallest value of 4.12 nm, further supporting the conclusion that fine sand contributes to pore structure refinement by reducing the presence of larger pores. A similar trend was observed in total porosity. At 90 days, Group A recorded the lowest porosity, reaching 5.02%, reflecting a highly compact matrix after extended hydration. In comparison, Group B showed a significant increase in porosity, reaching 9.79%, along with evident interfacial crack propagation as confirmed by SEM analysis. Group C maintained a substantially lower porosity of 7.40%, demonstrating the effectiveness of fine sand in limiting porosity development and improving structural density during long-term hydration.

The pore volume data showed consistent patterns. Group A, the pure paste, exhibited the lowest long-term pore volume at 0.0172 mL/g, indicating superior structural compactness. Group B, the standard sand mortar, showed a higher pore volume of 0.0336 mL/g at 90 days, suggesting a looser interfacial structure, which aligns with the results of pore size distribution analysis. Group C, containing fine sand, showed a continuous decrease in pore volume over time, reaching 0.0254 mL/g, confirming the sustained micro-filling effect of fine sand in optimizing the internal pore network. Median pore diameter data further supported these trends. Group A maintained a stable value around 67 nm throughout curing, indicating a uniform and compact pore structure. In contrast, sand-containing mortars exhibited smaller median pore diameters, particularly Group C, which decreased to 11 nm by 90 days. This substantial reduction highlights the critical role of fine sand in refining average pore size and enhancing the uniformity of the pore structure.

### 3.5. Fractal Characteristics

**Figure 12:**
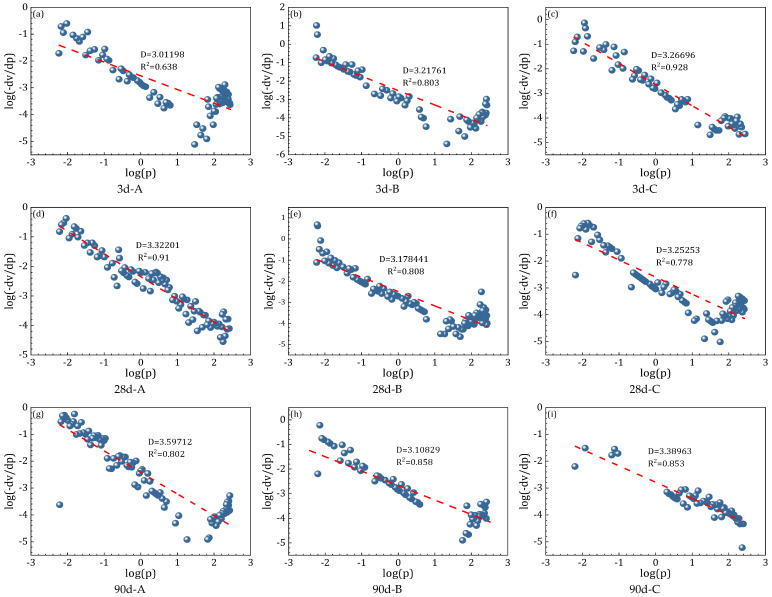
The fractal dimension of alkali-activated cementitious materials.

Given the complexity, irregularity, and randomness of the internal pore structure in alkali-activated cementitious materials, applying fractal theory to analyze pore size distribution provides valuable insight into microstructural characteristics and their relationship with macroscopic performance. Based on the MIP test results, the fractal properties of the pore structures were further examined to deepen the understanding of this correlation. Figure 12 presents the calculated fractal dimensions for each specimen group.

This figure illustrates the fractal characteristics of pore structures in specimens with different compositions across various curing ages. A higher fractal dimension (D) reflects a more complex and compact pore network and is generally positively correlated with improved macroscopic mechanical performance. As such, the fractal dimension provides a quantitative measure of pore structure complexity and offers meaningful insight into the density and evolution of the microstructure over time.

Although Group A exhibited a slightly lower fractal dimension compared to Groups B and C, it achieved the highest early compressive strength of 75.07 MPa, presenting an apparently contradictory trend. This can be attributed to the rapid and extensive formation of calcium–aluminosilicate hydrate gels during early hydration in the pure slag system. These abundant gel products effectively filled capillary pores and sealed microvoids, resulting in a dense matrix with strong mechanical integrity. Consequently, despite a lower fractal dimension, the pore network in Group A demonstrated high compactness and efficient stress transfer capability. This indicates that early-age strength is primarily governed by the rate and continuity of gel formation, rather than the overall geometric complexity of the pore structure. Therefore, the fractal dimension alone may not fully capture the mechanical performance of alkali-activated systems at early stages. In contrast, Groups B and C, which incorporated sand, exhibited more interfacial pores and relatively looser early-age structures, leading to reduced strength despite slightly higher fractal dimensions.

At 28 days, the fractal dimensions of all three groups increased, reaching 3.33201 for Group A, 3.59712 for Group B, and 3.17844 for Group C. Group B exhibited the highest fractal dimension at this stage, suggesting that the incorporation of standard sand enhanced the complexity and uniformity of the pore structure to some extent. However, its compressive strength remained lower than that of Group A, with values of 80.9 MPa and 97.5 MPa, respectively. This discrepancy indicates that despite the increased fractal dimension, weak interfacial bonding continued to restrict strength development in Group B. In contrast, Group C demonstrated consistent improvement in both the fractal dimension and compressive strength. This trend highlights the beneficial role of fine sand, whose smaller particle size and pronounced micro-filling effect contributed to a denser and more cohesive paste–aggregate interface, thereby enhancing structural compactness and mechanical performance.

At 90 days, the fractal dimension of Group A slightly decreased to 3.17844, while its compressive strength continued to increase, reaching 114.85 MPa—substantially higher than the other two groups. This suggests that long-term strength development was primarily driven by ongoing hydration and the continuous accumulation of gel products, despite the slight coarsening of the pore structure. Meanwhile, Group C exhibited an increase in the fractal dimension to 3.38963, accompanied by a compressive strength of 95.36 MPa, which notably surpassed Group B’s 89.59 MPa. This further confirms the effectiveness of fine sand in optimizing the pore network and enhancing the density of the interfacial transition zone during extended curing, thereby promoting sustained mechanical performance.

Comprehensive analysis indicates that Group A, consisting of a pure slag system, exhibited superior density and compressive strength across all curing ages, with a distinct advantage at early stages. However, the high slag content raises concerns regarding resource consumption and associated carbon emissions. In contrast, Group C achieved a 5.6% reduction in slag content compared to Group A, demonstrating considerable potential for lowering environmental impacts such as CO_2_ emissions. According to standard estimates, the partial replacement of slag with fine sand can proportionally reduce CO_2_ emissions, underscoring the environmental benefits and supporting the development of more sustainable AASC formulations. Groups B and C, both employing partial slag substitution with sand, not only contributed to environmental improvements but also maintained favorable long-term performance. Notably, Group C demonstrated synergistic growth in both the fractal dimension and compressive strength, reflecting enhanced structural compactness and strong potential for practical engineering applications. From the dual perspective of mechanical performance and environmental sustainability, Group C represents an optimized mix design strategy that effectively balances strength development with a reduced environmental footprint.

## 4. Conclusions

This study systematically examined the strength evolution, pore structure, microstructural development, and fractal characteristics of alkali-activated slag cementitious materials. The main conclusions are as follows:

The compressive strength of all groups increased significantly with extended curing time. Group A exhibited the highest strength at all ages, reaching 75.07 MPa at 7 days, 97.5 MPa at 28 days, and 114.85 MPa at 90 days, highlighting the high early reactivity and long-term densification capability of pure slag under alkaline activation.

Although the sand-incorporated Groups B and C showed lower strength at early ages compared to Group A, both exhibited continuous strength development over time. At 90 days, Group C surpassed Group B with a compressive strength of 95.36 MPa versus 89.59 MPa, confirming that the micro-filling effect of fine sand significantly improved paste densification and interfacial strength in the long term.

Pore structure analysis revealed that all specimens experienced progressive densification with curing age. Group A achieved high overall compactness but showed slight pore coarsening at later ages. In contrast, Groups B and C demonstrated continuous pore structure refinement, with Group C showing marked reductions in total porosity and large pore proportions—highlighting the structural optimization benefits of fine sand with smaller particle size.

Fractal dimension analysis further validated the correlation between pore structure complexity and mechanical performance. At 3 days, Group A had a lower fractal dimension yet exhibited the highest strength due to abundant early gel formation. Fractal dimensions increased for all groups by 28 days and showed minor fluctuations by 90 days. Group C, in particular, exhibited a sustained increase and stabilization in the fractal dimension, reflecting consistent long-term pore structure optimization aligned with strength improvement.

From the combined perspective of mechanical performance and environmental sustainability, Group A delivered excellent strength but involved high slag content and resource consumption. In contrast, Groups B and C, through partial slag replacement with sand, achieved environmental benefits while maintaining good long-term performance. Group C, in particular, demonstrated superior structural compactness, sustained strength development, and greater practical applicability, offering a more balanced and sustainable AASC formulation.

In summary, the incorporation of fine sand effectively improved the long-term pore structure and mechanical performance of alkali-activated slag cementitious materials while reducing slag consumption, thereby offering notable environmental benefits. The fractal dimension proved to be a valuable structural parameter for quantifying pore complexity and compactness, providing a reliable theoretical basis for the performance evaluation and sustainable mix design of alkali-activated materials.

## Figures and Tables

**Table 1:** Chemical composition of slag powder (%).

Component	SiO_2_	Al_2_O_3_	Fe_2_O_3_	CaO	MgO	SO_3_	MnO	TiO_2_	LOI
Slag powder	33.23	17.76	0.416	37.17	7.53	3.10	0.404	0.992	—

**Table 2:** Chemical composition of water glass (%).

Solid Content	Na_2_O	SiO_2_
36.07	8.64	27.43

**Table 3:** Mix proportions of alkali-activated slag cementitious material specimens (g).

Group	Slag Powder	Fine Sand	Standard Sand	Water Glass	Water	Total Water
A	400	0	0	136	40	120
B	450	0	1350	153	135	225
C	427.5	22.5	1350	146	130	216

**Table 4:** Pore structure parameters of alkali-activated slag cementitious material specimens.

Specimen	Threshold Pore Size (nm)	Most Probable Pore Size (nm)	Total Porosity (%)	Pore Volume (ml/g)	Median Pore Size (nm)
3d-A	1625.23	5.85	17.37	0.0595791	66.17
3d-B	2255.16	4.27	8.80	0.030184	9.1
3d-C	2339.15	4.59	8.11	0.0278173	9.4
28d-A	1415.26	4.29	7.83	0.0268569	67.52
28d-B	2439.94	4.25	9.299	0.03189557	10.7
28d-C	3473.03	4.22	8.33	0.0285719	14.3
90d-A	2843.1	4.23	5.02	0.0172186	66.8
90d-B	7382.83	4.16	9.79	0.0335797	20.7
90d-C	1877.2	4.12	7.40	0.025382	11

## Data Availability

The original contributions presented in this study are included in the article. Further inquiries can be directed to the corresponding authors.
